# Innovations in denture marking: Forensic applications and clinical implications: A review

**DOI:** 10.6026/9732063002001452

**Published:** 2024-11-30

**Authors:** Ajimol Theresa Jose, Tejaspreet Kaur, Sibani Sarangi, Sania Khan, Shubham Tripathi, Sooriaprakas Chandrasekaran, Ritik Kashwani

**Affiliations:** 1 Department of Prosthodontics & Implantology, AIMST University, Kedah, Malaysia; 2 Department of Conservative Dentistry and Endodontics , SKSS dental college, Sarabha, Ludhiana, India; 3 Department of Periodontology, Hitech Dental College and Hospital, Bhubaneswar, India; 4 Department of Orthodontics & Dentofacial Orthopedics, District Bhoj Hospital, Dhar, Indore, Madhya Pradesh, India; 5 Department of Conservative Dentistry and Endodontics, District Bhoj Hospital, Dhar, Indore, Madhya Pradesh, India; 6 Department of Conservative Dentistry and Endodontics, Meenakshi Ammal Dental College and Hospital, Meenakshi Academy of Higher Education and Research (Deemed to be University) Chennai; 7 Department of Oral Medicine and Radiology, School of Dental Sciences, Sharda University, Greater Noida, Uttar Pradesh, India

**Keywords:** Denture markers, forensics, inclusion, recognition, surface

## Abstract

Denture marking is a vital tool in forensic odontology and clinical practice, aiding in the identification of dental prostheses, especially in disaster victim identification and cases where traditional methods fail. Techniques are categorized into surface methods like engraving and laser etching and inclusion methods such as embedding RFID tags or barcodes. These markers provide reliable identification, assist in recovering lost prostheses and can be implemented at low cost in routine dental practice. Advancements like RFID technology have made denture marking more efficient and reliable. Overall, denture marking enhances patient safety, reduces redundancy and holds significant medico-legal value.

## Background:

The approach of integrating or inscribing personally identifiable data on or within a dental prosthesis typically removable denture for the intent of individual recognition is known as denture marking. It can be particularly beneficial for post-mortem authentication in dental forensics and additionally in healthcare settings in order to recover missing dentures with their true owners. Denture marking optimizes care for patients and enables disaster victim identification (DVI) both for legal and healthcare reasons [1].

## Denture markings include two major varieties:

## Surface and inclusion techniques:

Although surface markings like engravings and embossments are apparent on a prosthesis surface, they are also susceptible to deterioration over time. Denture bases are capable of being embedded with identification materials including microchips, barcodes, or metallic labels. Such methods provide an additional durable and impervious type of identification. The inclusion approach has become increasingly common considering its longevity and reliability, particularly with incorporating digital technology like Radio Frequency Identification (RFID) [2]. Traditional denture marking techniques entail the use of onion sheets and acetates owing to the ease of usage and convenience in clinical settings. During the production step of the denture, onion paper a thin, translucent material can be incorporated into the resin made from acrylic and imprinted with patient data, involving their name or identification number [3]. This method allows patients to get positive identification at an affordable price and with considerable ease. However, with time, especially when subjected to the moist environment of the oral cavity, the onion sheets are susceptible to wear, discoloration and deterioration that might compromise their long-term endurance [4].

Corresponding to onion sheets, acetates are transparent or clear sheets that are more durable. Print or write patient information on them to incorporate them into the denture base. In contrast to onion sheets, acetates have superior mechanical resistance and are less likely to deteriorate [5]. Onion sheets and acetates should be firmly embedded in the acrylic to preserve the prosthesis' biocompatibility and guard it from saliva [6]. These techniques offer a simple and inexpensive way to distinguish between dentures, but they are not quite as advanced or lasting as advanced systems like metallic labels or RFID chips; thus, they are more prone to wear down as time passes [7].

## There are two main types of denture markers:

Inclusion methods and surface marking methods. Each type of marker includes a distinct set of strategies. Employing surface marking techniques, identification is put directly onto the denture's surface, which renders it noticeable but susceptible to deterioration over time [8]. Methods like engraving, laser etching, dye stamping and scribing fall within this group of skills. While laser etching uses laser beams for higher accuracy, mechanical engraving etching transfers data onto the acrylic surface. Sharp tools are used to chisel information in scribing, while ink or dye is applied in dye stamping-a shorter-lasting approach [9]. On the contrary, inclusion techniques offer more strength and counterfeit resistance by integrating markers within the base of dentures while manufacturing. It involves fibre tags, lenticular lenses, barcodes, microchips, metallic labels and photographic markers [10]. Photographic markers contain laminated patient photos, whilst metallic labels consist of titanium or stainless steel. Barcodes and microchips (like RFID chips) convey electronic scanning and enhanced data storage. Given the angle at which they're viewed, lenticular lenses display distinct images. Fibre tags, on the flip side, are less costly options that embed medical information in resin. The robustness, intricacy and appropriateness of all methods for future identification differ [11].

## Methodology:

**Figure 1:**
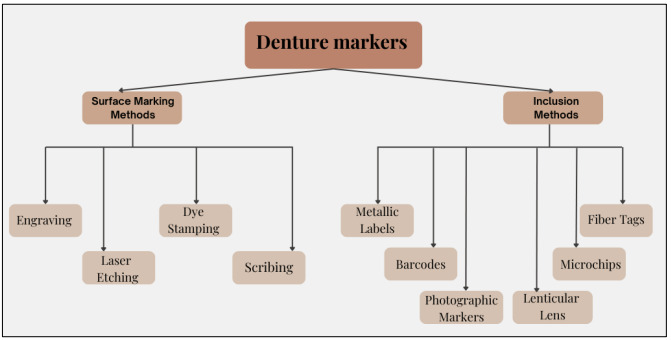
Prisma flowchart of the study

This study employs a comprehensive review of existing literature on denture marking techniques and their applications in forensic odontology and patient care. The primary objective is to assess the effectiveness, durability and practicality of various denture marking methods, including surface and inclusion techniques, in both clinical and forensic settings. The Prisma Flowchart of the study is shown in Figure 1.

## Literature search:

An extensive literature search was conducted using databases such as PubMed, Scopus and Google Scholar to identify relevant studies on denture marking. The keywords used included "denture markers," "forensic dentistry," "surface marking techniques," "inclusion methods," and "RFID in dentures." Articles published between 2000 and 2024 were reviewed, focusing on both historical and recent advancements in denture marking technology.

## Selection criteria:

Studies were selected based on their relevance to denture marking techniques, forensic applications, patient safety and medico-legal implications. Only peer-reviewed articles in English were included. Studies focusing on the use of innovative materials such as RFID tags, metallic labels and laser etching in dentures were prioritized to evaluate contemporary practices. Prisma flowchart of the study is shown in Figure 1.

## Data extraction:

## Data were extracted on the following:

The methodology for this study involves conducting a detailed literature review to assess the effectiveness and practical applications of various denture marking techniques. Key focus areas include comparing surface and inclusion methods, analyzing their durability and longevity and evaluating their forensic and clinical utility. Special attention is given to the cost-effectiveness and ease of integrating these techniques into routine dental practice. Additionally, the review examines the medico-legal implications, including issues of patient privacy and ethical concerns, particularly in the context of forensic identification and healthcare settings. The study seeks to provide a comprehensive understanding of how these methods function both in practical and medico-legal scenarios.

## Analysis:

The extracted data were analyzed to compare the advantages and disadvantages of each method. Particular attention was given to the durability of denture markers, especially in harsh conditions such as disaster scenarios (*e.g.*, fire, decomposition). The review also examined the ease of integrating denture markers with electronic health records (EHRs) and their role in preventing denture misplacement in healthcare settings like nursing homes.

## Ethical considerations:

The methodology also includes a discussion of ethical issues related to patient privacy, informed consent and the possible implications of denture marking in medico-legal contexts. This section reviews how different regions address these concerns and the variations in legal frameworks governing denture marking.

## Limitations:

The study acknowledges the limitations, such as variability in reporting standards for the durability of denture markers and limited access to data on the use of newer technologies like RFID in certain geographic regions. This methodology facilitates a comprehensive understanding of how denture markers function as tools for patient safety and forensic identification.

## Review:

In scenarios concerning accidents, natural disasters, or in medical and residential organizations, denture markings are a vital tool for forensic identification and safeguarding patients. Dentists may ensure that dentures may be traced down to their first proprietor by precisely embedding identification data, like a patient's name or ID number, in the base of the denture. This is particularly crucial for the elderly, who suffer from dementia, or those in nursing homes who may be lost or perplexed [13]. Denture markers can be affixed via surface engraving or newer techniques with durability and longevity, like the inclusion of microchips or metallic labels. Denture markings offer perks, yet the usage differ across the globe: certain regions use them, whereas some don't. It is also essential to address moral dilemmas, such as patient privacy and approval and to make certain individuals are versed in the process. Denture marking is an evolving method which offers even more reliability and safety in the forensic and medical sectors as technology develops [14]. Denture markings have grown into a crucial process that serves several functions, chief amongst which is identification of patients. Denture labeling, or the procedure of placing markers in dentures, is an enduring yet growing discipline with significant implications for patient safety, health care management and forensic studies [15].

The selection of appropriate sites for placing denture markers is crucial to ensure their durability, functionality and minimal interference with the prosthesis' aesthetic and mechanical properties. The most commonly recommended site is the posterior palatal area of maxillary dentures and the lingual flange of mandibular dentures [16]. These locations provide adequate space for embedding the marker without compromising the structural integrity of the denture. Additionally, these areas are less prone to wear and tear during mastication, thus preserving the longevity of the marker. The buccal flange since it's got sufficient surface space; both upper and lower dentures ought to be given consideration. Yet, care must be taken to avoid excess prominence that might harm the prosthesis's comfort or fit [8].

Another suitable location is the base of dentures in the molar or premolar region as it is less susceptible to direct stress and is less apparent to the patient [17]. Marker placement in the posterior seal area Maxillary dentures proves helpful as they are shielded from mechanical stresses while biting and are undetectable from the outside. The dimension of the marker and the way it impacts the look and function of the denture must be taken into account while selecting its position to prevent compromising the prosthesis's stability, retention, or occlusal balance [18].

## Discussion:

Denture marking is justified in dentistry due to its critical role in patient identification, forensic odontology and prosthetic management. It ensures positive identification of prostheses, especially in cognitively impaired or institutionalized patients, reducing the risk of denture misplacement or swapping. In forensic cases, such as disaster victim identification (DVI), denture markers aid in identifying remains when other methods are not viable. Additionally, denture markers provide legal documentation, safeguarding against malpractice and ensuring accountability in prosthetic care. The use of RFID chips, metallic labels, or barcodes enhances patient safety and the integration of electronic health records (EHRs), streamlining dental care and identification processes [19, 20, 21- 22]. Denture markings can erode over time, depending on factors such as the marking technique, the material of the denture and environmental exposure. Methods like superficial engraving are more prone to wear compared to deeper or embedded techniques like laser etching or embedding metal. Dentures, typically made of acrylic resin, are subjected to mechanical forces during daily use, such as chewing and cleaning, which can cause the markings to gradually wear down. Additionally, exposure to harsh cleaning agents, extreme temperatures, or chemical disinfectants can accelerate the degradation of both the denture material and the markings. Improper application of markings, especially if they are not well-sealed or embedded deeply enough, May also leads to faster erosion. Therefore, choosing durable marking techniques and proper application are essential for long-lasting identification on dentures. The following table illustrates the advantages and disadvantages of denture markers.

As an effective and lasting method for accurate identification of both living and deceased people, denture marking is vital in medico-legal scenarios, particularly in forensic odontology, security of patients and identity. Denture markers have become crucial for disaster victim identification (DVI) of forensics, especially if victims are subjected to harsh circumstances like fire, decomposition, or trauma that might compromise traditional determining methods like fingerprint or DNA analysis. When linked with patient-specific data, dentures may act as vital identifiers since they're heat-resistant and often stay attached following such events. Legally, this applies significantly in scenarios of massive casualties, criminal probes and cases of unidentified corpses when denture markings could represent the sole way of authentication [20]. Denture markers are employed in hospitals, particularly in geriatric care, to prevent prosthetic misplacement or inadvertent denture swapping. These are all prevalent issues in social contexts like nursing homes. Protecting patient and doctor safety is feasible via identifiable markers as evidence of ownership in lost or missing dentures. Denture markers also help with the proper detection and continuation of care in situations, including patients with cognitive impairments like dementia or Alzheimer's disease, which reduces the likelihood of medical malpractice claims [21]. Legally speaking, if a denture marker with identifiable data is present, it may also serve as documentation in malfeasance or prosthetic mishandling cases, proving that a denture was created especially for the recipient. This shields dentists from claims of carelessness or stolen identity. Denture markers offer undisputed, tamper-proof records that are immune to legal inspection and help authenticate cases involving missing people, inheritance conflicts and claims for insurance [17, 18, 19- 20]. The reliability and precision of denture marking in medico-legal settings further enhanced with the emergence of digital recognition tools, such as RFID chips or scan able barcodes. This has made it possible to incorporate denture marking effectively into forensic databases and guarantees conformity to clinical as well as regulatory requirements. Denture labeling is thus essential in preserving patient safety, transparency and legal integrity in both forensic and medical contexts. Its medico-legal significance goes beyond mere identification [22].

## Conclusion:

To sum up, denture markers were vital for prosthetic dentistry to enhance criminal prosecution and security for patients. In clinical and forensic events, integrating them into dental prostheses-whether via surface marks or advanced inclusion techniques like RFID chips and barcodes-ensures robust and reliable authentication. It not only lowers the risk of device being lost in susceptible groups, such as elderly or cognitively impaired patients, yet it provides vital evidence for disaster victim identification (DVI) and litigation. Denture marking therefore offers on-going advantages in dentistry and forensic science by acting as a crucial tool for ensuring both clinical accountability and medico-legal protection.

## Figures and Tables

**Figure 2:**
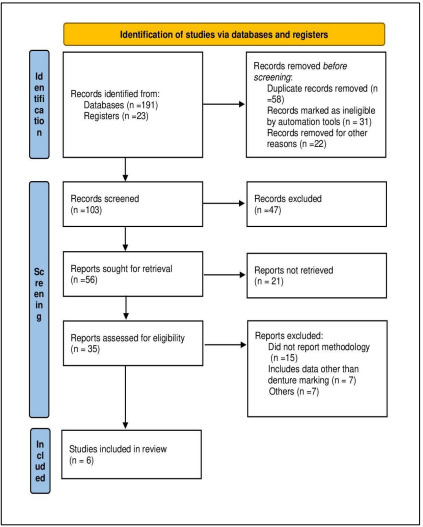
Classification of denture markers

**Table 1:** Advantages and disadvantages of denture markers

**Aspect**	**Advantages**	**Disadvantages**
Forensic Identification	Facilitates identification of victims of disasters (DVI) and other positive identification in forensic situations.	Ineffectual in instances where dentures fall apart or are lost altogether.
Durability	Embedded markers, such as RFID chips and metallic labels, are resilient to adverse conditions and wearing.	Surface marks are susceptible to fading, abrasion and corrosion.
Patient Safety	Ensuring proper recognition is essential for medical care, especially for geriatric or mentally ill individuals.	Prosthesis fit and ease of use could be impaired by faulty placement.
Medico-Legal Protection	Safeguards against fraud and incorrect identification by providing evidence in legal circumstances.	Requiers patient autorisation, posing concerns with aesthetics and confidentiality.
Technological Integration	Advanced markers (*e.g*, RFID, barcodes) allow electronic health record (EHR) integration and efficient tracking.	More complex markers increase costs and require specialized equipment for integration.
DVI: Disaster Victim Identification
RFID: Radio Frequency Identification
EHR: Electronic Health Record

**Table 2:** Literature review of the study

**AUTHOR**	**YEAR**	**TITLE**	**JOURNAL**	**OBJECTIVE**	**METHODOLOGY**
Mohan. J *et al.* [1]	2012	"Denture marking" as an aid to forensic identification	J Indian Prosthodont Soc	To explore denture marking as an aid in forensic identification.	Discusses the significance of denture marking in identifying individuals and reviews various methods used.
Datta. P *et al.* [2]	2010	The various methods and benefits of denture labelling	J Forensic Dent Sci	To review different methods of denture labelling and their forensic advantages.	Overview of existing denture labelling methods and a discussion on their importance in forensic science.
Bansal. PK *et al.* [3]	2011	Denture labelling: A new approach	Contemp Clin Dent	To propose a novel approach to denture labelling for forensic identification.	Presents an innovative technique for denture labelling and its application in human identification.
Colvenkar. S *et al.* [4]	2022	A Novel Denture Labelling Technique for Human Identification	Cureus	To introduce a novel denture labelling technique and assess its utility.	Describes a new method for denture labelling and its practical application in forensic human identification.
Mahoorkar. S *et al.* [8]	2013	Denture identification using unique identification authority of India barcode	J Forensic Dent Sci	To introduce the use of Indian unique identification barcodes for denture marking.	Proposes a unique method of using barcodes from the Indian UID system for denture identification.
Kareker N. *et al.* [12]	2014	A Review on Denture Marking Systems: A Mark in Forensic Dentistry	J Indian Prosthodont Soc	To review existing denture marking systems used in forensic dentistry.	Comprehensive review of various denture marking systems, highlighting their strengths and limitations.
